# Integrating patient reported measures as predictive parameters into decisionmaking about palliative chemotherapy: a pilot study

**DOI:** 10.1186/s12904-016-0101-z

**Published:** 2016-03-01

**Authors:** Anna Creutzfeldt, Anna Suling, Karin Oechsle, Anja Mehnert, Djordje Atanackovic, Melanie Kripp, Dirk Arnold, Alexander Stein, Julia Quidde

**Affiliations:** Department of Oncology, Hematology, BMT with Section Pneumology, University Medical Center Hamburg-Eppendorf, Hubertus Wald Tumour Center - University Cancer Center Hamburg, Martinistr. 52, 20246 Hamburg, Germany; Department of Medical Biometry and Epidemiology, University Medical Center Hamburg-Eppendorf, Martinistr. 52, 20246 Hamburg, Germany; Department of Oncology, Hematology, BMT with Section Pneumology, University Medical Center Hamburg-Eppendorf, Martinistr. 52, 20246 Hamburg, Germany; Department of Medical Psychology and Medical Sociology, University Medical Center Leipzig, Philipp-Rosenthal-Straße 55, 04103 Leipzig, Germany; Department of Oncology, Hematology, BMT with Section Pneumology, University Medical Center Hamburg-Eppendorf, Martinistr. 52, 20246 Hamburg, Germany; Department of Oncology/Hematology, University Hospital Mannheim, University of Heidelberg, Theodor-Kutzer-Ufer 1-3, 68167 Mannheim, Germany; Tumour Biology Center Freiburg, Breisacher Straße 117, 79106 Freiburg, Germany; Department of Oncology, Hematology, BMT with Section Pneumology, University Medical Center Hamburg-Eppendorf, Hubertus Wald Tumour Center - University Cancer Center Hamburg, Martinistr. 52, 20246, Hamburg, Germany; Department of Oncology, Hematology, BMT with Section Pneumology, University Medical Center Hamburg-Eppendorf, Hubertus Wald Tumour Center - University Cancer Center Hamburg, Martinistr. 52, 20246, Hamburg, Germany

**Keywords:** Symptom Burden, Quality Of Life, Tumour Response, Progression Free Survival, Cancer

## Abstract

**Background:**

Systemic treatment has proven to improve physical symptoms in patients with advanced cancer. Relationship between quality of life (QoL) or symptom burden (SYB) and treatment efficacy (tumour response and survival) is poorly described. Therefore, we evaluated the predictive value of pretreatment QoL and SYB on treatment outcomes.

**Methods:**

Eligible patients had metastatic gastrointestinal cancers and were about to receive 1st/2nd line palliative chemotherapy. 47 patients were consecutively enrolled. QoL and SYB were assessed by EORTC QLQ-C30 and MSKCC MSAS questionnaires before treatment and after first response evaluation after 8–12 weeks. Logistic regression analysis of QoL and SYB for prediction of objective treatment efficacy was performed. Patients were categorized according to response rate (RR) based on RECIST1.1 and progression free survival (PFS). PFS was categorized by a ratio (individual PFS/expected PFS) in above median (ratio ≥ 1) or below median PFS (ratio < 1). QoL and SYB were analysed for RR groups (partial response, stable or progressive disease) and PFS ratio (PFSR).

**Results:**

Objective response to chemotherapy and increase in PFS were associated with better pretreatment QoL and less SYB. Patients with future objective treatment efficacy (PFSR ≥ 1) evidenced clinically relevant better role/emotional/cognitive/social functioning and less fatigue and appetite loss at baseline in comparison to PFSR < 1 (>10 points difference). Lowest scores in all functioning scales at treatment start were seen in patients with future PFSR < 1. Global health status (EORTC), PSYCH subscale and global distress index (MSAS) predicted PFSR, even if adjusted for gender, age, cancer type, ECOG and line of treatment (*p <* 0.05). Interestingly, improved QoL and SYB (subjective benefit) were noted even in patients with worse pretreatment status and no objective tumour response.

**Conclusion:**

Future non-responders seem to show distinct QoL patterns before chemotherapy. This may facilitate early detection of patients deriving less or even no benefit from treatment regarding prolongation of survival. Even in patients with primarily progressive disease QoL and SYB may improve during treatment. Integration of QoL and SYB assessment into decision-making about palliative chemotherapy seem to be an important approach to improve patient outcome and should be further evaluated.

## Background

Gastrointestinal cancers are among the most prevalent causes of tumour-related deaths worldwide [1, 2]. Disease management in the palliative setting has dramatically improved throughout the last decade with the implementation of individualized and multimodal treatment approaches and a large number of new licensed drugs and integrated treatment modalities [3–9]. Moreover, predictive molecular markers (e.g. RAS mutational status in metastatic colorectal cancer) enable improved patient allocation [10]. However, despite the current developments, the majority of patients will receive palliative treatment in order to improve or delay symptoms and prolong overall survival. Despite using patient and tumour related factors (e.g. co-morbidity, age, histology) for treatment selection, a substantial proportion of patients will not derive a relevant benefit from palliative systemic treatment. Identifying these patients before or at least early during palliative chemotherapy is of utmost importance.

Currently, several scores are available to determine individual prognosis of patients based on upfront biochemical markers and performance status [11–14]. Although prognostic and predictive variables differ between tumour entities, performance status and/or age are included in the majority of scores. However, performance status according to Eastern Cooperative Oncology Group (ECOG) is a mix of tumour related symptom burden and patient related pre-”cancer” co-morbidity and/or frailty and does not allow precise evaluation of reasons for future non-response to treatment.

Quality of life (QoL) and symptom burden (SYB) can be easily assessed by standardized self-administered questionnaires like the European Organisation for Research and Treatment of Cancer Quality of Life Questionnaire (EORTC QLQ-C30) or the Memorial Sloan Kettering Cancer Center Symptom Assessment Scale (MSKCC MSAS) and give a more specific overview of the current patient status. Likely, QoL and SYB have an influence on the course of disease and potentially on treatment efficacy. Although assessment of QoL has been commonly applied in current trials, only little is known so far about the impact of QoL and SYB prior to chemotherapy on treatment efficacy defined by key oncological trial parameters such as response rate (RR) or progression free survival (PFS) [15–17].

The purpose of this study was to investigate the influence of QoL and SYB prior to chemotherapy on treatment efficacy (RR and PFS) determined by radiological assessment (adjusted for established prognostic factors, e.g. performance status (PS) and age). This exploratory trial was designed to answer the following questions:

1. Do QoL and SYB prior to chemotherapy predict treatment efficacy in terms of RR and PFS and do they allow the identification of future non-responders?
2. How do QoL and SYB change during chemotherapy considering the achieved tumour response?

Given the exploratory and descriptive nature of these questions, no formal statistical assumptions were determined. To evaluate the predictors among patients‘ QoL and SYB for treatment efficacy we used logistic regression models.

## Methods

### Patient selection

Patients were eligible if standard (in terms of intensity and dosing) first- or second-line palliative chemotherapy for histologically confirmed metastatic gastrointestinal cancer was scheduled. The applied (standard) regimens are recorded in Table 1. The follow-up assessment had to take place after first response evaluation after 8–12 weeks. To avoid selection bias patients were consecutively recruited. Participants had to be able to complete a battery of self-administered questionnaires. Minimum age was 18 years. All patients provided written informed consent before study entry according to institutional regulations. The ethical review committee of the medical association of Hamburg had approved the trial.

**Table 1:** Expected progression free survival and applied regimen in the current study

	1st line chemotherapy		2nd line chemotherapy	
Cancer site	Regimen (reference)	Expected PFS [months]	Regimen (reference)	Expected PFS [months]
Colon/rectum	FOLFOX + bevacizumab or cetuximab [34, 35]	9	FOLFIRI+/− panitumumab or bevacizumab [36–38]	5
Pancreas	Gemcitabine based [39, 40]	4	fluoropyridimine +/− oxaliplatin [41]	2
Stomach/esophagus	fluoropyrimidine/platinum +/− trastuzumab for HER2 positive [42–44]	6	taxane or irinotecan [45]	3
Anal	5FU/platinum [46]	6	-	-
Cholangiocarcinoma	Gemcitabine +/− Cisplatin [47]	6	-	-

Patients were consecutively recruited in the outpatient clinic of the Department of Oncology, Hematology and Bone Marrow Transplantation, University Medical Center Hamburg-Eppendorf during daily routine of chemotherapy visits from April 2012 to August 2013.

### Assessments

QoL and SYB were assessed at two time points**:** at the beginning of any new first- or second-line chemotherapy and after the discussion of the first radiographic evaluation of tumour response (usually performed after 8–12 weeks after the first cycles’ beginning onwards).

The patients’ performance status at time of trial inclusion were graded according to ECOG performance status [18]. Furthermore, the following baseline demographics were assessed: age, gender, participation period, and line of treatment. Tumour measurements using either computed tomography (CT) or magnetic resonance imaging (MRI) of all tumour lesions were performed to evaluate RR (according to RECIST version 1.1) as well as PFS [19]. In order to assess potential influence of dose modifications on treatment efficacy these data were obtained at the first radiographic evaluation.

To evaluate QoL and SYB, we used the standardized and well-established EORTC QLQ-C30 as well as the validated Memorial symptom assessment scale (MSKCC MSAS) [20, 21].

### Objective treatment efficacy

Objective treatment efficacy was evaluated according to response criteria in solid tumours (RECIST) version 1.1. [19]. Following these revised guidelines, we differentiated between partial response (PR), progressive disease (PD), and stable disease (SD).

Furthermore, PFS (in months) was defined as the time period between start of chemotherapy and either disease progression according to RECIST v1.1 or death. All scores were calculated at baseline and follow-up according to a PFS ratio (PFSR) categorizing patients into those achieving an expected or above median PFS for their respective disease and treatment line (PFSR ≥ 1) and patients with a below median PFS (PFSR < 1). The ratio was determined by the individual “achieved” PFS divided by the “expected” PFS. The “expected” PFS values were derived from the available literature taking into account current clinical trials and are displayed in Table 1.

### EORTC QLQ-C30

The EORTC Quality of Life Questionnaire (EORTC QLQ-C30) was used to assess health-related quality of life and was evaluated by following the procedures suggested in the Scoring Manual [22, 23]. This questionnaire is composed of five functioning scales (physical, social, role, cognitive, and emotional functioning), a scale for global quality of life, three 3-symptom scales (fatigue, nausea and vomiting, and pain), and a number of single items assessing additional symptoms (dyspnea, insomnia, appetite loss, constipation, diarrhea, and financial difficulties). All scales and single-item measures are linearly transformed to scores from 0 to 100. On the function scales and global quality of life scale, a score of 100 represents maximum functioning, whereas on the symptom scales and single items, a score of 100 indicates the worst possible symptoms. The interpretation of the patient groups’ mean values of the EORTC QLQ-C30 was performed regarding the previously reported threshold of a 10-point difference as clinically relevant [24].

### MSKCC-MSAS

The MSKCC-MSAS [25] assesses the prevalence, severity, and distress of 32 symptoms experienced during the prior week. Symptom prevalence and severity are measured on a four-point Likert scale. The prevalence is classified as “rarely“, “occasionally“, “frequently“and “almost constantly“. Symptom severity is defined as “slight“, “moderate“, “severe“ and “very severe“.

Distress caused by a symptom is measured on a five point Likert scale ranging from “not at all“ to “very much“ (in-between: “a little bit“, “somewhat“, “quite a bit“).

The questionnaire’s evaluation was performed based on the authors’ guidelines [25].

Following these guidelines, a score was calculated for each symptom and different symptom scores were combined to calculate different subscale scores: PSYCH-subscale (feeling sad, worrying, feeling irritable, feeling nervous, difficulty sleeping and difficulty concentrating), PHYS-subscale (lack of appetite, lack of energy, pain, feeling drowsy, constipation, dry mouth, nausea, vomiting, change in taste, weight loss, feeling bloated and dizziness), Global Distress Index (GDI (feeling sad, worrying, feeling irritable, feeling nervous, lack of appetite, lack of energy, pain, feeling drowsy, constipation, dry mouth)) and MSAS total score (all 32 symptom scores).

### Statistical analysis

To evaluate potential predictors among patients’ QoL and SYB prior to chemotherapy on future PFSR, logistic regression models were used. Due to the likely influence of variables like ECOG performance status, regression analyses were adjusted for these parameters. Overall, 19 linear regression analyses for all QoL and SYB variables were conducted, each one adjusted for gender, age, cancer site, ECOG performance status and line of treatment. The dependent variable was PFSR (PFSR ≥ 1 and PFSR < 1). Significance was set at *p =* 0.05. Odds ratios (OR) are presented with 95 % confidence limits (CI).

All statistical analyses were performed using SPSS 20 (IBM Corp.).

## Results

### Patient characteristics and treatment efficacy

Overall, 47 patients completed the first questionnaires of whom 40 patients (85.1 %) completed the follow-up questionnaires. Patient characteristics are summarized in Table 2. Colorectal, pancreatic and gastric cancer were the most frequent tumour types in this cohort.

**Table 2:** Patients’ characteristics

Characteristics	Total (%) *n =* 47 (100)
Mean age in years (SD)	61.7(9.5)
Gender	
Female	18 (38.3)
Male	29 (61.7)
Chemotherapy	
First-line chemotherapy	36 (76.6)
Second-line chemotherapy	11 (23.4)
Cancer site	
Colon/rectum	18 (38.3)
Pancreas	12 (25.5)
Stomach/esophagus	13 (27.6)
Anal	2 (4.3)
Cholangiocarcinoma	2 (4.3)
ECOG Performance Status	
0	6 (12.8)
1	29 (61.7)
2	12 (25.5)
RECIST	
Partial Remission	16 (34)
Stable Disease	22 (46.8)
Progressive Disease	9 (19.2)
PFS-Ratio	
≥ 1	30 (63.8)
< 1	17 (36.2)
Mean participation period in weeks (SD)	8.8 (3.5)

PR according to the RECISTv1.1 was detected in 16 patients (34 %), SD in 22 (46.8 %), and PD in 9 patients (19.2 %). According to the PFS ratio, 30 patients (63.8 %) showed a PFS of the expected median or above (PFSR ≥1), whereas 17 patients (36.2 %) showed a PFS below the expected median (PFSR < 1).

Dose modifications occurred in overall 9 patients (3 treatment delays for a maximum of 8 days, 6 dose reductions by a maximum of 25 % for all active agents). However, treatment efficacy was similar in patients with dose modifications (4 patients with PR, 3 with SD and 2 with PD).

### Quality of life and symptom burden in relation to objective treatment efficacy

Baseline and follow-up QoL and SYB mean values were interpreted for PFSR and RR. The scores of the baseline EORTC QLQ-C30 and MSKCC MSAS prior to chemotherapy illustrated remarkable differences between patients with future responding and future non-responding tumours. Patients with future treatment efficacy (PFSR ≥ 1) had a clinically relevant better Qol/GHS, role functioning (RF), emotional functioning (EF), cognitive functioning (CF) and social functioning (SF) in comparison to PFSR < 1 (>10 points). Lowest scores in all functioning scales at treatment start were seen in patients with future PFSR < 1 and PD. The GHS prior to chemotherapy had a significant impact (*p =* 0.006) on PFSR and may be seen as a predictive parameter for treatment efficacy (Table 3). Similar albeit non-significant results were seen in the PR group compared to PD. Moreover, fewer symptoms prior to chemotherapy may predict treatment efficacy. With a difference of 10 points the PFSR ≥ 1 group showed less fatigue and appetite loss, and with a difference of 5 points less dyspnoea and financial problems. The chance to belong to the PFSR < 1 group changes with a one unit increase of GHS by the factor of 0.95. Thus, a 10 points higher score at baseline would be associated with a change in the odds by the factor 0.61 (0.95 to the power of 10 = 0.61) (*p =* 0.006, OR = 0.95, 95 % CI 0.92-0.99).

**Table 3:** Logistic regression analysis on baseline variables Dependent variable: PFSR. Independent variables: EORTC and MSKCC SAS variables adjusted for gender, age, cancer type, ECOG performance status and line of treatment

Independent Variable	p-value	Regr.	OR	95 %-CI for OR	
				min	max
EORTC QLQ C30					
Global Health Status	0.006	−0.050	0.951	0.918	0.986
Physical Functioning	0.465	−0.009	0.991	0.967	1.015
Role Functioning	0.360	−0.009	0.991	0.973	1.010
Emotional Functioning	0.130	−0.017	0.983	0.961	1.005
Cognitive Functioning	0.139	−0.025	0.976	0.944	1.008
Social Functioning	0.099	−0.016	0.984	0.965	1.003
Fatigue	0.219	0.014	1.014	0.992	1.037
Nausea and Vomiting	0.938	−0.001	0.999	0.972	1.027
Pain	0.962	0.001	1.001	0.980	1.022
Dyspnoea	0.851	0.002	1.002	0.984	1.019
Diarrhoea	0.109	−0.020	0.980	0.956	1.005
Insomnia	0.860	0.002	1.002	0.984	1.020
Appetite Loss	0.197	0.011	1.011	0.995	1.027
Constipation	0.331	−0.014	0.986	0.957	1.015
Financial Difficulties	0.214	0.012	1.012	0.993	1.032
MSKCC-MSAS					
PSYCH-Subscale	0.032	0.896	2.450	1.081	5.556
PHYS-Subscale	0.283	0.514	1.671	0.654	4.272
GDI	0.014	1.120	3.064	1.256	7.475
Total Score	0.405	0.532	1.702	0.487	5.953

GHS improved during course of chemotherapy particularly in patients achieving disease control (PR and SD). Interestingly, even in patients with no or decreased impact on survival (PFSR < 1) mean GHS improved by more than 10 points. The functioning scales showed no remarkable changes at follow up assessment for PFSR ≥ 1, whereas PFSR < 1 showed improved SF. RF deteriorated in the PFSR < 1 group potentially reflecting patients’ limitation in everyday life due to the harmful combination of chemotherapy’s side effects and progressive tumour disease. Symptom scores as fatigue, pain and insomnia at follow-up assessment remarkably deteriorated in the PD group.

The GDI seems to have predictive value for treatment efficacy in terms of PFSR (*p =* 0.014, OR = 3.064), even if adjusted for age, gender, cancer site, line of treatment and ECOG PS. Further more, the PSYCH-Subscale seems to have a predictive value for the future response to chemotherapy (*p =* 0.032, OR = 2.450). PHYS-Subscale shows similar trends. Prior to chemotherapy, high mean values and therefore poor physical and psychological functioning (PHYS- and PSYCH Subscale) were seen in the PD group as well as for PFSR < 1. During course of chemotherapy, PHYS and PSYCH subscales indicated improved symptom control and less psychological problems in all patients independent from RR or PFS. However, the improvement was pronounced in those patients starting with worse QoL and SYB and deriving less benefit in terms of survival (PFSR < 1). GDI and MSAS total score at follow up showed similar changes. Interestingly, even patients with PD showed this improvement of physical and psychological symptoms in the MSAS.

**Table 4:** EORTC QLQ-C30 mean values at baseline and follow-up assessment of response rate and progression free survival-ratio according to RECIST (PR = partial remission, SD = stable disease, PD = progressive disease, PFS = progression free survival ratio)

	Response			PFS		
	RECIST	Baseline	Follow-up	Ratio	Baseline	Follow-up
QoL/GHS (Quality of Life/Global Health Status)	PR	53.65	57.81	>1	59.17	56.85
	SD	53.03	55.00			
	PD	41.67	36.11	<1	36.76	49.24
PF (Physical Functioning)	PR	65.00	70.83	>1	68.22	69.20
	SD	70.91	67.30			
	PD	57.04	48.89	<1	62.75	62.42
RF (Role Functioning)	PR	57.29	48.96	>1	57.78	55.75
	SD	57.58	54.76			
	PD	37.04	22.22	<1	46.08	34.85
EF (Emotional Functioning)	PR	70.31	68.23	>1	65.00	66.96
	SD	59.85	63.33			
	PD	43.52	55.56	<1	51.96	59.09
CF (Cognitive Functioning)	PR	87.50	81.25	>1	88.33	83.33
	SD	88.64	83.33			
	PD	68.52	72.22	<1	77.45	77.27
SF (Social Functioning)	PR	39.58	51.04	>1	52.77	57.14
	SD	56.06	64.17			
	PD	31.48	22.22	<1	33.33	51.52
FA (Fatigue, Symptom Score)	PR	37.50	47.22	>1	44.44	43.68
	SD	50.00	41.27			
	PD	62.96	74.07	<1	54.90	52.52
NV (Nausea and Vomiting, Symptom Score)	PR	17.71	13.54	>1	16.67	14,94
	SD	16.67	15.08			
	PD	16.67	5.56	<1	17.65	10.61
PAIN (Symptom Score)	PR	29.17	30.21	>1	26.67	21.83
	SD	21.97	15,87			
	PD	35.19	50.00	<1	27.45	30,30
DYS (Dyspnoe, Symptom Score)	PR	20.83	31.25	>1	33.33	37.93
	SD	39.39	44.44			
	PD	51.85	55.56	<1	39.22	45.45
INS (Insomnia, Symptom Score)	PR	27.08	22.92	>1	37.78	28.74
	SD	50.00	39.68			
	PD	33.33	44.44	<1	41.18	45.45
AL (Appetite Loss, Symptom Score)	PR	31.25	33.33	>1	32.22	34.48
	SD	31.82	28.57			
	PD	62.96	44.44	<1	47.06	24.24
CON (Constipation, Symptom Score)	PR	14.58	6.25	>1	14.44	9.52
	SD	9.09	11.67			
	PD	12.50	0.00	<1	6.25	6.06
DIA (Diarrhoea, Symptom Score)	PR	18.75	27.08	>1	28.89	29.76
	SD	27.27	26.67			
	PD	29.63	33.33	<1	17.65	21.21
FIN (Financial Difficulties, Symptom Score)	PR	29.17	27.03	>1	22,22	27.16
	SD	24.24	35.09			
	PD	18.52	0.00	<1	29.41	33.33

**Table 5:** MSKCC-MSAS mean values at baseline and follow-up assessment of response rate and progression free survival ratio according to RECIST (PR = partial remission, SD = stable disease, PD = progressive disease, PFS = progression free survival)

	Response			PFS		
	CT-scan	Baseline	Follow-up	Ratio	Baseline	Follow-up
PSYCH (Subscale MSAS)	PR	0.93	0.86	>1	0.95	0.87
	SD	1.10	0.97			
	PD	1.57	1.27	<1	1.47	1.14
PHYS (Subscale MSAS)	PR	0.99	1.03	>1	0.92	0.96
	SD	0.90	0.84			
	PD	1.34	1.14	<1	1.19	0.89
GDI (Global Distress Index, MSAS)	PR	1.24	1.21	>1	1.24	1.23
	SD	1.42	1.30			
	PD	2.03	1.98	<1	1.89	1.52
Total Score MSAS	PR	0.78	0.85	>1	0.81	0.87
	SD	0.84	0.85			
	PD	1.15	0.96	<1	1.00	0.83

## Discussion

The current study evaluated the role of QoL and SYB at baseline and the changes after the first response assessment by CT or MRI in patients with metastatic gastrointestinal cancer. Particularly the prediction of treatment efficacy determined by key oncological parameters (tumour response and PFS) and the alteration of QoL and SYB after chemotherapy were assessed.

The results illustrate that patients who will achieve a relevant tumour response or at least an expected PFS (PR and PFSR ≥ 1) had a significantly better health status (GHS), less psychological problems (PSYCH-Subscale) and lower distress (GDI) prior to chemotherapy than patients who progressed early. Therefore, GHS, PSYCH-Subscale and GDI seem to have predictive value for treatment efficacy. All other items of the applied questionnaires showed a similar association of better QoL and SYB with increased treatment efficacy (PR and PFSR ≥ 1). However, the number of patients was too small to show a significant association for further single items or sub-scales with treatment efficacy. Although the analyses were adjusted for ECOG performance status and line of therapy these findings could be influenced by disease burden and aggressiveness. Thus, future studies should focus on single tumour types in prior untreated patients including established scores determining biology of disease and co-morbidity [11–14].

The patients with low QoL and high SYB prior to chemotherapy seem to be at high risk not to radiographically respond to chemotherapy in comparison to patients with moderate SYB and relatively good QoL. In particular, suffering of physical symptoms (mainly fatigue and appetite loss) prior to chemotherapy may indicate a lack of future objective treatment efficacy. Interestingly, these factors were among the main issues of relevance for cancer patients in a recent survey [26]. The impact of SYB before chemotherapy on treatment efficacy is well known and reflected by the prognostic value of performance status on survival, which has therefore been integrated in different scores in the last decades [11–14, 27–29]. Moreover, physical symptoms at baseline significantly predicted treatment efficacy in metastatic gastrointestinal cancer [12]. Relevant physical symptoms such as fatigue and appetite loss may reveal an aggressive or advanced disease prior to chemotherapy and could thus allow the early detection of high-risk patients for future non-response [30, 31].

Despite the severity of physical symptoms in patients with a future non-responding tumour disease, several interesting issues were noted regarding psychosocial factors. The future non-responding patients as compared to the future responders were more limited in role- and emotional functioning and had higher distress levels.

During the course of chemotherapy, stabilisation or even improvement of the initial low QoL and high SYB (subjective benefit) is of high relevance in a palliative setting independent of objective tumour response or prolongation of survival. Interestingly, patients with worse QoL and SYB at baseline as compared to those with moderate scores seem to derive more subjective benefit from palliative chemotherapy in terms of improvement of quality of life and symptom burden. This points towards a beneficial impact of palliative chemotherapy even though no objective tumour response or increase in survival is achieved [32]. Of note, the current study did only evaluate pretreatment QoL and SYB and 8–12 weeks thereafter. Thus, long term effects, as the recently shown deleterious effects of chemotherapy use on QoL at the end of life, could not be analysed [33]. The noted short term subjective benefit despite radiographical non-response in patients with worse pretreatment QoL and SYB might be caused by at least some effect on tumour growth or by the psychological effect of applying an active and potentially effective treatment.

Some patients do not derive any benefit (subjective or objective) at all by palliative chemotherapy. In the subgroup of patients with progressive disease at first follow up pain, fatigue and insomnia increased accompanied by remarkable deteriorations of SF, RF and GHS during chemotherapy. Premature stop of chemotherapy and initiation of palliative care would likely improve QoL and SYB in this patient group. Although, QoL and SYB prior to chemotherapy seem to predict objective treatment efficacy in terms of RR and PFS, stratification of patients deriving subjective benefit (defined as improvement of QoL and SYB) is difficult. Further research is needed to potentially define a cut off value of a composite of several QoL and SYB items or subscores to upfront identify patients with a detrimental impact of palliative chemotherapy. The current study underlines the potential utility of integrating patient reported measures into decision-making about palliative chemotherapy.

This study has several limitations. Incomplete follow-up data pose a problem, single centre evaluation as a well as the small sample size. Seven patients were lost to follow-up due to progressive disease or death. However, based on the above mentioned findings inclusion of more patients would probably have enhanced the findings, but not relevantly changed them. With the inclusion of different tumour types and first as well as second line patients the population is rather heterogeneous. Therefore, the logistic regression analyses were adjusted for gender, age, cancer type, line of treatment and in particular ECOG performance status. Patients were recruited consecutively, thus selection bias was minimized. Patients’ selection was based on a scheduled standard chemotherapy for the respective disease setting in terms of intensity and dosing. Therefore, patients scheduled for upfront dose reduction or single agent treatment due to reduced general health status were not eligible. In order to assess the potential influences of dose modifications on treatment efficacy these data were obtained at the follow up imaging, showing no relevant correlation. To be able to set the PFS data obtained in the heterogeneous study population into context, the PFS ratio was chosen for further analyses. The expected PFS data are derived from patient populations included into clinical trials with inclusion criteria differing from our patient population in some aspects (e.g. ECOG mainly limited to ≤1), whereas particularly the median age was in the range of the trial selection criteria. This study was a pilot study to generate hypotheses to be prospectively tested in future sub-projects on QoL and SYB from ongoing clinical trials in better defined patient populations with specific survival data. In consideration of testing multiple hypotheses results should be interpreted with caution. Interestingly, the global scores were significantly altered, whereas most single items showed strong trends. This distribution points to clinically relevant and reliable results. Nevertheless further research is urgently warranted focusing on distinct disease entities and settings in larger groups of patients.

## Conclusion

In cancer patients, physical and mental condition will influence the impact of palliative chemotherapy on objective (RECIST) and subjective (alleviation of quality of life and symptoms) treatment efficacy. A good quality of life, less psychological problems and lower distress are suggested to be positive predictors for treatment efficacy in terms of objective tumour response and survival. In contrast, subjective benefit could be achieved even in patients with low quality of life and high symptom burden prior to chemotherapy. Although further research is needed to better define patients’ subgroups, integration of QoL and SYB assessments into individualized decision-making in the palliative setting seems to be of high relevance.
